# Defining Low Prognosis Patients Undergoing Assisted Reproductive Technology: POSEIDON Criteria—The Why

**DOI:** 10.3389/fendo.2018.00461

**Published:** 2018-08-17

**Authors:** Sandro C. Esteves, Matheus Roque, Giuliano M. Bedoschi, Alessandro Conforti, Peter Humaidan, Carlo Alviggi

**Affiliations:** ^1^ANDROFERT, Andrology and Human Reproduction Clinic, Campinas, Brazil; ^2^Department of Surgery, University of Campinas (UNICAMP), Campinas, Brazil; ^3^Faculty of Health, Aarhus University, Aarhus, Denmark; ^4^ORIGEN, Center for Reproductive Medicine, Rio de Janeiro, Brazil; ^5^Division of Reproductive Medicine, Department of Gynecology and Obstetrics, University of São Paulo, Ribeirão Preto, Brazil; ^6^Department of Neuroscience, Reproductive Science and Odontostomatology, University of Naples Federico II, Naples, Italy; ^7^Fertility Clinic Skive Regional Hospital, Skive, Denmark

**Keywords:** assisted reproductive technology, hypo-responder, low responder, ovarian stimulation, poor ovarian response, poor ovarian reserve, POSEIDON criteria

## Abstract

Women with impaired ovarian reserve or poor ovarian response (POR) to exogenous gonadotropin stimulation present a challenge for reproductive specialists. The primary reasons relate to the still limited knowledge about the POR pathophysiology and the lack of practical solutions for the management of these conditions. Indeed, clinical trials using the current standards to define POR failed to show evidence in favor of a particular treatment modality. Furthermore, critical factors for reproductive success, such as the age-dependent embryo aneuploidy rates and the intrinsic ovarian resistance to gonadotropin stimulation, are not taken into consideration by the current POR criteria. As a result, the accepted definitions for POR have been criticized for their inadequacy concerning the proper patient characterization and for not providing clinicians a guide for therapeutic management. A novel system to classify infertility patients with “expected” or “unexpected” inappropriate ovarian response to exogenous gonadotropins—the POSEIDON criteria—was developed to provide a more nuanced picture of POR and to guide physicians in the management of such patients. The new standards are provoking as they challenge the current terminology of POR in favor of the newly defined concept of “low prognosis.” This article provides readers a critical appraisal of the existing criteria that standardize the definition of POR and explains the primary reasons for the development of the POSEIDON criteria.

## Introduction

The primary goal of assisted reproductive technology (ART) is the birth of a healthy child. This outcome depends on a multitude of non-mutual independent factors, including female age and the effect of ovarian stimulation (OS) (1, 2). Nowadays, clinicians rely on patient characteristics, ovarian reserve markers, and treatment history—if available—for clinical decision-making concerning OS strategy, aiming at securing the shortest time to live birth as well as the lowest risk of complications (3, 4).

The number of oocytes retrieved after OS represents a critical cornerstone of ART since it is an independent predictor of the likelihood of pregnancy (5–7). Although the ideal number of oocytes collected after ovum pickup has been a matter of debate in recent years, it seems reasonable to define a typical ovarian response as the retrieval of 10–15 oocytes after conventional OS (5). However, a significant proportion of patients who undergo OS has either a poor (<4 oocytes) or suboptimal (4–9 oocytes) number of oocytes retrieved (3–9). As a consequence, the number of resulting embryos available for transfer or cryopreservation is reduced, thus jeopardizing treatment success (3, 4, 10–12). The cost of *in vitro* fertilization (IVF) tends to be higher in poor and suboptimal responders than in normal responders because different strategies or repeat treatment cycles might be required. Altogether, these factors cause emotional, physical, and financial distress for the couple, particularly when multiple treatment cycles are required.

The standards that define poor ovarian response (POR) vary widely as several factors either isolated or in combination are used for identification of such patients (13). Not surprisingly, the reported prevalence of POR fluctuates markedly between 5.6 and 35.1% (14, 15). Regardless of the chosen definition, it is clear that the POR population accounts for a substantial subset of women treated in IVF clinics nowadays (16). Driven by socioeconomic and other issues, many women are currently postponing motherhood which results in a higher number of patients seeking ART treatments in their late thirties and early forties. Women in this age range are more likely to have a diminished ovarian response due to natural aging of the ovaries, highlighting the need for particular attention to this group of women undergoing ART (17).

The central element in the pathophysiology of low ovarian response is the presence of a reduced number of follicles responsive to FSH. This phenomenon is most often found in women of advanced maternal age, mainly because of reduced ovarian reserve caused by accelerated follicular loss (18). In some cases, however, a low ovarian response might be seen in good ovarian reserve patients caused by a suboptimal gonadotropin dosage used for OS, for example in obese women (19), or due to the presence of genetic polymorphisms affecting endogenous gonadotrophins or their receptors (20–22). Both conditions ultimately alter the response of recruitable follicles to exogenous gonadotrophins (23–25). It is, therefore, clear that the so-called POR does not have a single cause. Indeed, the population with a diminished ovarian response is heterogeneous and sometimes difficult to characterize (14).

Most women diagnosed as poor responders are less likely to conceive or might even have their IVF cycle canceled due to lack of embryos for transfer (26). Nonetheless, some studies evaluating this patient population report reasonable cumulative pregnancy rates, ranging from 6 to 47% after three cycles, according to patient's age (27). Moreover, up to 40% of women who respond poorly in their first IVF cycle, as defined by the number of oocytes collected, have been reported to end up as normal responders in the second cycle (11, 16, 26). These figures indicate that not all women diagnosed with low ovarian response are similar regarding the likelihood of pregnancy. The optimal portrayal of this group of women with a low ovarian response is essential for proper counseling regarding the chances of pregnancy and the use of individualized strategies to increase IVF success (3, 4). Nevertheless, the current definitions for POR have been criticized for their inadequacy concerning a proper characterization of the POR population and for not providing clinicians a guide for therapeutic management (3, 4, 9, 14, 15). In this review, we provide an overview of existing criteria utilized to define the POR population, along with their advantages and shortcomings. Subsequently, we discuss the issues of ovarian resistance to gonadotropin stimulation and the importance of balancing quantity and quality with regard to oocytes retrieved. Lastly, we explain why a novel system for the identification and classification of low prognosis patients undergoing ART—the so-called POSEIDON criteria—was developed.

## Criteria for the definition of poor ovarian response to ovarian stimulation

Several standards have been developed for the definition of POR. Parameters related to patient demographics, ovarian reserve tests, and outcomes of previous IVF cycles—alone or combined—are used to define the POR population (Table 1) (28–49). The numerous existing definitions differ concerning the parameters utilized and the threshold values established for each criterion. In a 2011 systematic review of 47 randomized clinical trials involving women with POR, 41 different definitions were used to define this group of patients (13). Notably, different definitions were used even in trials by the same group of researchers and no more than three trials use the same definition. In this review, the authors observed that the age criterion—considered essential by some investigators for the description of POR—was used in only 9% of studies (13). The disparity in POR definition renders the interpretation of trial results challenging. At the very least, conclusions about the different interventions tested must be interpreted with caution as regards their application in clinical practice.

**Table 1:** Parameters used isolated or in combination to define the poor ovarian response patient.

**Characteristics**	**Parameter**	**References**
Demographics	Female age	(28)
Ovarian reserve markers	Antral follicle count	(29, 30)
	Basal serum FSH levels	(31–33)
	Serum anti-Müllerian hormone levels	(30)
Previous IVF cycle outcomes	History of cycle cancelation	(34, 35)
	Number of preovulatory follicles on day of trigger	(28, 33, 35–41)
	Serum estradiol levels on day of trigger	(32, 37, 39, 42, 43)
	Number of oocytes retrieved	(34, 37, 43)
	Number of mature oocytes retrieved	(44, 45)
	Number of good quality embryos	(46)
	Daily and total gonadotropin consumption	(47–49)

Various terminologies utilized to define this group of patients further reflect the discrepancy of the definition of the POR patient. Researchers and clinicians often use ambiguous terms as POR, low ovarian response (47, 50, 51), hypo-response (20, 21), and diminished ovarian reserve (52–54). According to a 2015 survey study among reproductive specialists, the most used criterion to define POR was “the number of follicles produced” (14), unlike the POR criteria used in research studies. To complicate matters further, a not-for-profit patient organization dedicated to providing education to couples suffering from infertility (https://resolve.org/) defines POR as those women who require large doses of medication and who make less than an optimal number of oocytes, meaning that patients themselves have introduced a new element into the already complicated POR equation, namely, the suboptimal response to ovarian stimulation.

## The bologna criteria

In 2011, the European Society of Human Reproduction and Embryology (ESHRE) carried out the first systematic effort to define women with inadequate response to OS (55). This consensus definition—known as the Bologna criteria—was initially introduced with the primary objective of standardizing the definition of the POR patient based on oocyte quantity for use in research studies. The authors made specific recommendations for investigators to avoid use of random definitions in prospective clinical trials or conduct meta-analyses including studies with distinct POR definitions (55).

According to Bologna criteria, at least two of the following three criteria must be present to classify a patient as poor responder, namely, (i) Advanced maternal age, (ii) Previous POR after OS, and (iii) Abnormal ovarian reserve tests (Table 2). The age of 40 years and retrieval of three or fewer oocytes were adopted as the cutoffs to discriminate women with and without POR. Ovarian reserve tests, namely antral follicle count (AFC) and anti-Müllerian hormone (AMH) levels were also included, with variable ranges of <5–7 follicles or <0.5–1.1 ng/ml, respectively.

**Table 2:** ESHRE Bologna criteria.

**PARAMETERS INCLUDED**
Advanced maternal age (≥ 40 years) or any other POR risk factor
A previous incident of POR (cycles canceled or ≤3 oocytes with a conventional ovarian stimulation protocol)
A low ovarian reserve test (AFC <5–7 follicles or AMH <0.5–1.1 ng/ml)
Two of these three criteria are required for a POR diagnosis. In addition, two previous episodes of POR after maximal stimulation are sufficient to classify a patient as POR even in the absence of the other criteria mentioned.

*POR, poor ovarian response; AFC, antral follicle count; AMH, anti-Müllerian hormone*.

The Bologna criteria were partially successful in its intended primary goal. Among 51 POR interventional trials registered in *clinicaltrials.gov* from July 2011 to March 2017, 23 (45%) adopted the Bologna criteria. The number of subjects enrolled in such trials varied markedly from 23 to 939, but the vast majority of trials were not powered to detect differences in pregnancy rates. In fact, a sample size of ~1,000 subjects would be required in binary outcome superiority trials to have a 90% chance of detecting, as significant at the level of 5%, a 20% increase in pregnancy rates between the control group and experimental group (https://www.sealedenvelope.com/power/binary-superiority/). Among the published trials with an adequate sample size to avoid a type II error (https://clinicaltrials.gov), only two reported a potential benefit of a given intervention with regard to pregnancy (56, 57).

A few retrospective cohort studies were also published using the Bologna criteria. On average, a live birth rate (LBR) of 10% or less was observed in women diagnosed with POR (58–60), therefore, suggesting that the Bologna criteria might be able to select a homogeneous population with poorer reproductive outcomes during ART. The correct identification of the subset of women with poor prognosis in IVF, apart from its usefulness in terms of clinical management and counseling, would be necessary from a public health perspective, particularly in countries with governmental treatment reimbursement (58).

## Limitations of the existing POR criteria

A review from 2016 accumulating the evidence of interventional clinical trials in POR revealed that over 90% trials were unable to detect meaningful differences in pregnancy rates (61). These disappointing results might be caused by the fact that the available studies used various POR definitions and suboptimal study designs, thus, making it difficult to draw valid conclusions for any given treatment strategy (62, 63).

Patient heterogeneity is deemed to be a significant shortcoming in studies evaluating strategies for POR, including those in which the Bologna criteria were applied (64). In a 2013 study, different LBRs were reported for Bologna POR aged ≤35 (12%), 36–39 (8%), and ≥40 (6%) (59). Likewise, Hu et al. retrospectively evaluated 592 IVF cycles in Bologna criteria PORs and reported that pregnancy outcomes varied according to age group (65). The authors showed that implantation rates ranged from 15.3 to 29.4% in patients under 35 years. By contrast, it ranged from 6.3 to 24.1% in patients ≥35 years. Along the same lines, Cohen and colleagues retrospectively assessed live birth rates in a large Bologna POR patient cohort aged 40 years or greater (16). The live birth per cycle was 3.3 times higher (11.61 vs. 3.54%, *P* < 0.001) in patients aged 40–43 with more than three oocytes compared to counterparts with less than three oocytes. Furthermore, a 2017 RCT evaluating the use of recombinant LH supplementation in Bologna criteria POR showed that—in a post-hoc analysis—the subset of patients classified as moderate or severe poor responders who received LH supplementation had higher LBR and lower pregnancy loss than the general population of POR patients (57).

Although the ESHRE consensus established the minimum criteria for the definition of POR, numerous patient categories with potentially different prognosis might be generated by using the criteria mentioned above (Table 3). Notably, studies explicitly evaluating pregnancy outcomes according to these subgroups of patients yielded conflicting results (58, 66, 67) (Table 4). Whereas reproductive success was similar among Bologna subgroups in the studies of Busnelli et al. (58) and La Marca et al. (66), the results differed according to the subset evaluated in the series of Bozdag et al. (67). In the latter study, which to our knowledge included the largest retrospective analysis of POR patients undergoing ART to date, the likelihood of pregnancy varied significantly according to the subgroups of POR evaluated (Table 4).

**Table 3:** Different patient categories generated by combining the parameters used to define the poor ovarian response patient according to Bologna criteria.

**Criteria**	**Combined with**
≥ 40 years	One previous POR episode Abnormal ORT
Other risk factor	One previous POR episode Abnormal ORT
One previous POR	≥40 years Other risk factor Abnormal ORT
Abnormal ORT	≥40 years Other risk factor Previous POR episode
2 previous episodes of POR after maximal stimulation	Alone Or with any other criteria

*POR, poor ovarian response (cycles canceled or ≤3 oocytes with the use of conventional ovarian stimulation); ORT, ovarian reserve tests (AFC <5–7 follicles or AMH <0.5–1.1 ng/mL); Other risk factor: genetic or acquired conditions possibly linked to a reduced number of resting follicles*.

**Table 4:** Clinical studies evaluating IVF outcomes in different subgroups of poor ovarian responders according to the Bologna criteria.

**Study**	**Number of patients (IVF/ICSI cycles) included**	**Subgroups included**	**Live birth rate/cycle (number of cycles)**	**Ability of Bologna criteria to identify homogeneous patient populations with similar pregnancy outcomes**
Busnelli et al. (58)	362 (362)	Group 1: anamnestic risk factors for POR and one episode of POR; Group 2: one previous episode of POR and abnormal ORT; Group 3: anamnestic risk factors for POR and abnormal ORT; Group 4: anamnestic risk factors for POR, one previous POR cycle and abnormal ORT Group 5: two episodes of POR after maximal stimulation	Group 1: 10% (40) Group 2: 4% (52) Group 3: 6% (190) Group 4: 8% (73) Group 5: 0% (7) *P-*values did not differ among subgroups (P = 0.65)	Yes; The study suffered from a type II error due to small patient cohort included in each subgroup.
La Marca et al. (66)	210 (452)	Group 1: ≥ 40 years-old + previous POR; Group 2: previous POR and abnormal ORT; Group 3: ≥ 40 years-old + abnormal ORT; Group 4: previous POR + ≥ 40 years-old + abnormal ORT; Group 5: two previous POR episodes	Group 1: 7.4% (76) Group 2: 6.6% (91) Group 3: 5.9% (76) Group 4: 6.7% (136) Group 5: 5.5% (73) *P*-values not provided	Yes; The study suffered from a type II error due to small patient cohort included in each subgroup.
Bozdag et al. (67)	821 (1257)	Group 1: ≥40 years-old + previous POR episode; Group 2: ≥40 years-old + AFC <7; Group 3: AFC <7 + previous POR episode; Group 4: ≥40y + AFC <7 + previous POR episode	Group 1: 3.3% (123) Group 2: 6.3% (253) Group 3: 8.7% (575) (*P* = 0.001; statistically different from all other groups) Group 4: 2.3% (306) (*P* = 0.002; statistically different from all other groups)	No; The number of subjects in each group was adequate to avoid a type II error.

*ORT, ovarian reserve test; Anamnestic risk factors: advanced maternal age (≥40years), evidence of ovarian endometrioma at the basal ultrasound, previous ovarian surgery, previous chemotherapy, genetic abnormalities, shortening of the menstrual cycle*.

Lastly, another limitation of the Bologna criteria relates to the biomarkers cut-offs used to classify POR patients. The ranges of 5–7 for AFC and, more importantly, 0.5–1.1 ng/ml for AMH seems quite wide. In fact, little information was provided by the authors of the ESHRE consensus about the accuracy of such ranges in predicting POR (55). Since the attributed importance of ovarian biomarkers is high, technical and performance characteristics should be considered when applying cut-off ranges, in particular, the lack of standardized methods for the assessment of ovarian reserve markers among centers (68).

## Ovarian resistance to exogenous gonadotropins: a previously neglected aspect

Ovarian stimulation is a crucial element of most IVF programs. The use of GnRH analogs in association with exogenous gonadotropins promote adequate follicular growth and steroidogenesis in the majority of normogonadotropic women who undergo ART. In the modern ART era, ovarian biomarkers, including AFC, and AMH have been used with fair accuracy to predict ovarian response to gonadotropin stimulation, thus, allowing clinicians to individualize OS (69). However, AFC and AMH cannot predict an unexpectedly poor or suboptimal response to gonadotropin therapy in women with adequate pre-stimulation parameters. Indeed, patients with adequate ovarian reserve might show hypo-responsiveness to gonadotropin stimulation (70, 71). The reasons for ovarian resistance to gonadotropin stimulation are not entirely understood. However, increasing evidence indicates that women with the so-called “hypo-response” to OS might harbor genetic mutations or single nucleotide polymorphisms (SNPs) of gonadotropins and their receptors that influence ovarian sensitivity to gonadotropin stimulation despite an apparently good prognosis (21, 25, 72–74).

Despite broadly categorized as PORs, the fate of women with hypo-response to OS differs from the classic POR patient. The results of a 2014 meta-analysis compiling 1129 IVF/ICSI cycles in POR patients supplemented or not with recombinant human LH (rec-hLH) illustrate this phenomenon (27). In this aforementioned review, the definition of POR to gonadotropin stimulation was based on the criteria utilized by each included study. It was noted that significantly more oocytes were retrieved in rec-LH supplemented cycles than in recombinant human FSH (rec-hFSH) monotherapy cycles (12 studies, *n* = 1077; weighted mean difference +0.75 oocytes; 95 % CI 0.14–1.36). The use of rec-hLH supplementation also improved clinical pregnancy rates by 30% overall (14 studies, *n* = 1179; relative risk [RR] 1.30; 95 % confidence interval [CI] 1.01–1.67; intention-to-treat population [ITT] population). Nevertheless, a careful examination of the included studies reveals that the beneficial effect of rec-hLH was more pronounced in studies involving hypo-responders rather than in those with classic POR. The inclusion of studies involving hypo-responders in that review explains the overall favorable results observed with rec-LH supplementation in the POR patient. Indeed, a 2018 systematic review carried out by the International Collaborative Group for the Study of rec-hLH (iCOS-LH) showed that a clear distinction between hypo-responders and classic PORs is paramount since the clinical relevance of adding rec-LH to OS was only evident in hypo-responders (75). Researchers have rightfully argued that critical methodological issues like the one discussed above should be taken into account when designing studies on poor responders (64, 76, 77).

From a clinical perspective, hypo-responders represent a patient category that differs from both normal responders and the classic POR. The hypo-responder is a patient with a normal ovarian reserve who ends up having an unexpected suboptimal or poor response to OS, usually manifested by a low follicular output rate (FORT), use of increased total dosages of gonadotropin, or lower than expected number of oocytes retrieved (9, 21, 25, 72). Management of hypo-responders might be associated with increased treatment costs, decreased cumulative live birth rates, and increased time to live birth. Until now, however, none of the POR criteria have taken into account this group of hypo-responders to ovarian stimulation.

## Oocyte quantity versus quality

The decline in fertility with aging is caused by both a progressive reduction in the primordial follicle number across the woman's lifespan as well as an increased rate of oocyte chromosomal abnormalities and cytoplasmic dysfunctions (18). These phenomena ultimately result in a reduction of oocyte quantity and quality, thus, explaining the poorer IVF outcomes in older women when compared to younger counterparts.

Data from large databases unequivocally show that IVF success depends on both the number of oocytes retrieved and the women's age (5, 6). The critical role of female age on oocyte quality is easily illustrated by comparing delivery rates according to age in women with similar oocyte yield (5, 6); in this scenario, the older the patient the lower the delivery rates. This effect is noted not only in the general infertile population, but also in poor responders (15).

Despite the overall notion that the prognosis of a patient undergoing IVF can be measured by the number of oocytes retrieved, a valid critique of Bologna criteria and other classification systems for POR is that these standards fail to identify young women with expected POR due to abnormal ovarian biomarkers; i.e., women below 35 years-old who have not undergone OS (78, 79). Preimplantation genetic studies using microarray-based comparative genomic hybridization and next-generation sequencing (NGS) show that embryo euploidy rates are markedly higher in women younger than 35 years of age than older counterparts (80, 81). In fact, embryo ploidy is probably the leading factor explaining the differences in success rates between younger and older women who undergo IVF (82).

The probability of achieving at least one euploid blastocyst for transfer in patients undergoing IVF increases as a function of blastocyst cohort size in all age categories (80, 81). Since blastocyst euploidy rates are independent of cohort sizes, the higher the number of oocytes retrieved the higher the probability of having an embryo cohort with at least one euploid embryo (80, 81). Therefore, oocyte quantity and the age-related embryo euploidy rate are essential aspects to consider for both counseling purposes and treatment planning in women with POR. Failure to include these aspects in clinical studies might result in stratification of women with distinct biological characteristics, a bias that could dilute the magnitude of the effect concerning the intervention studied.

## A plea for a more optimal definition and stratification of the low responder patient undergoing ART: the poseidon criteria

Despite the advancement toward a better definition of the POR patient with the publication of the Bologna criteria in 2011 (55), little has been achieved in terms of clinical guidance concerning management. To date, clinicians remain without evidence-based guidance for therapeutic management of the POR patient and often rely on personal experience or anecdotal facts to handle such patients (14). Thus, development of criteria aiming at identifying and stratifying patients with low prognosis in ART is of utmost importance for clinical management. A correct stratification of homogeneous groups of low prognosis women could also help researchers identify treatment strategies best suited for each patient category.

**Figure 1:**
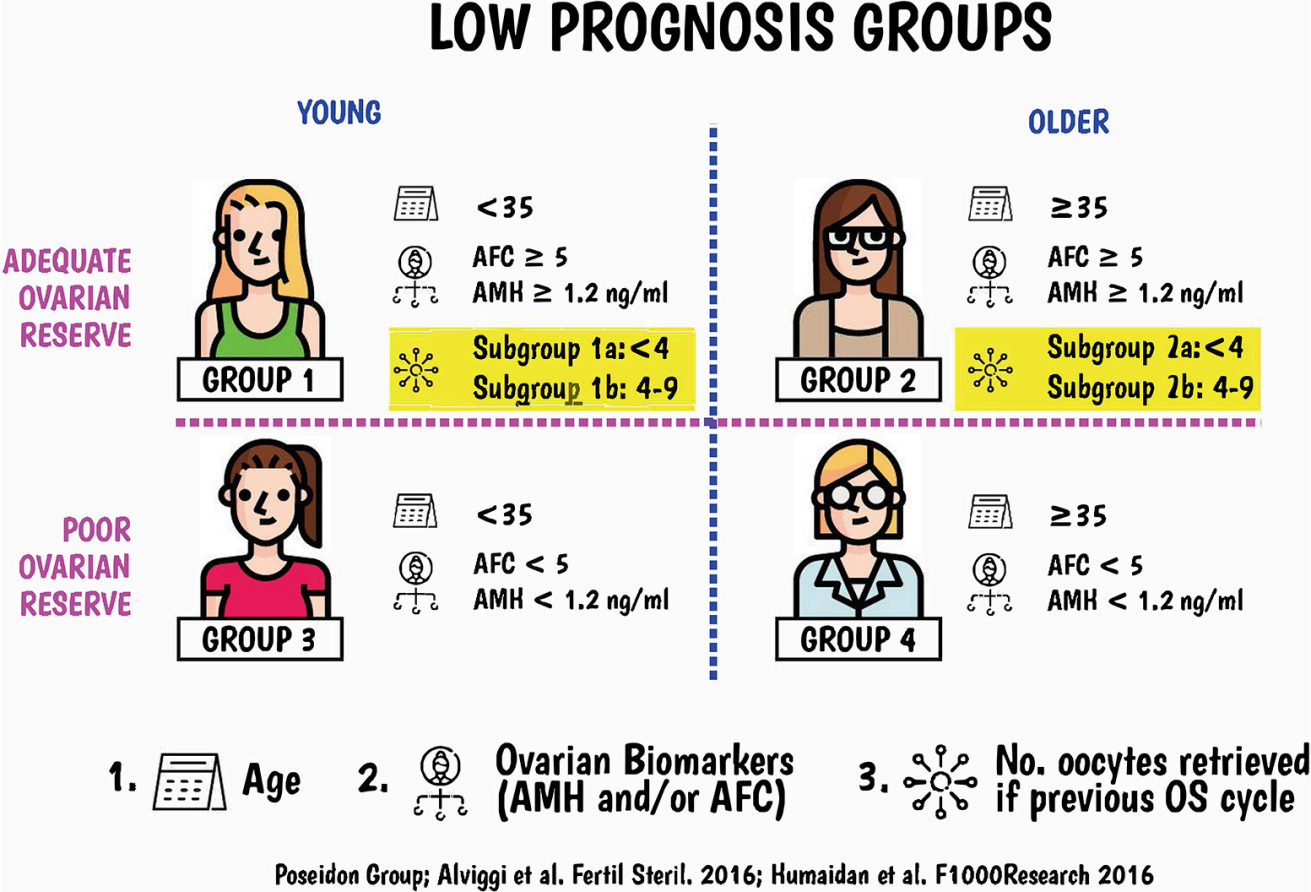
The new Poseidon criteria to identify and stratify infertility patients with “expected” or “unexpected” impaired ovarian response to exogenous gonadotropins undergoing ART. Four distinct groups of low prognosis patients can be established based on quantitative and qualitative parameters, namely: 1. The age of the patient and the expected embryo aneuploidy rate; 2. Ovarian biomarkers [antral follicle count [AFC] and/or anti-Müllerian hormone [AMH]], and 3. The ovarian response of the patient in terms of oocyte quantity provided a previous cycle of stimulation was carried out. Art drawing by Chloé Xilinas, EXCEMED, Rome, Italy.

The recently established POSEIDON (**P**atient-**O**riented **S**trategies **E**ncompassing **I**ndividualize**D O**ocyte **N**umber) Group, a collaborative effort among clinicians and researchers with a particular interest in reproductive endocrinology and ART, proposes a new and more detailed stratification of low prognosis patients who undergo OS for IVF (83, 84). A series of articles within this research topic of Frontiers in Endocrinology will discuss in great detail the newly launched POSEIDON criteria. In brief, this new system aims to introduce a fine-tuning of POR, using clinically relevant criteria to guide the physician (Figure 1). Essentially, the POSEIDON group proposes a change in the definition of POR from quite heterogeneous criteria to the concept of low prognosis, which better reflects the reproductive potential of these patients.

“Low Prognosis” seems to be the ideal terminology because it allows not only to identify patients who have a reduced probability of pregnancy in ART, but also to stratify the low prognosis patients into distinct categories based on quantitative and qualitative parameters, namely: (i) The age of the patient and the expected embryo aneuploidy rate; (ii) Ovarian biomarkers, and (iii) The ovarian response of the patient provided a previous cycle of stimulation was carried out (83). In addition to providing a system for the identification and classification of low prognosis patients undergoing ART, the group introduced a new measure of clinical success, namely, the ability to retrieve the number of oocytes needed to obtain at least one euploid blastocyst for transfer in each patient (84).

Notably, the POSEIDON group does not advocate trial-and-error to identify patients classified as groups 1 and 2. Other published algorithms might be considered as a means to optimize oocytes yield on the first cycle (85). However, the information from a previous cycle should be used wisely, whenever available, to most optimally plan the next ovarian stimulation strategy.

The POSEIDON criteria allow the clinician to first of all classify patients who have low prognosis in ART and secondly to prepare a stimulation plan aiming at reaching the number of oocytes needed to obtain at least one euploid blastocyst for transfer (4, 86). It is anticipated that the new concept of low prognosis will help improve the management of patients undergoing ART, promote a tailored approach to patient handling, and identify more homogeneous populations for clinical trials, thereby, providing better tools with which to maximize IVF success rates.

## Conclusions

Management of patients with an impaired ovarian reserve or POR to exogenous gonadotropin stimulation has challenged reproductive specialists for several decades. Apart from our limited understanding of its pathophysiology, wide heterogeneity exists in the definition of POR. A critical shortcoming of the existing POR criteria, which is largely based on ovarian biomarkers and numbers of oocytes retrieved after OS, is that they group women with distinct clinically relevant characteristics. This could explain the lack of scientific evidence to support any effective intervention for POR patients. As a result, practitioners have utilized different strategies in clinical management—often not evidence-based—since none of the existing POR criteria provide a clear path for management. In practical terms, counting the number of oocytes retrieved or estimating such numbers using ovarian biomarkers is not enough for clinical management. Equally important is the ability to determine the ovarian sensitivity to gonadotropins, which is modulated by genetic factors involving both gonadotropins and their receptors, and the age-related decrease in oocyte quality which largely depends on chromosomal abnormalities occurring before meiosis II.

The POSEIDON (**P**atient-**O**riented **S**trategies **E**ncompassing **I**ndividualize**D O**ocyte **N**umber) group—founded in 2015- introduced a new system to stratify infertility patients with “expected” or “unexpected” impaired ovarian response to exogenous gonadotropins. Furthermore, the group proposed a new measure for successful ART treatment, namely, the ability to retrieve the number of oocytes necessary to obtain at least one euploid embryo for transfer in each patient. This new stratification aims at providing a more nuanced picture of POR using clinically relevant criteria to guide the physician in the management of this increasing group of patients. Thus, the POSEIDON group proposes a change in the definition of POR, with sub-grouping, resulting in more homogenous populations. Hopefully, this new classification system will prove to be of daily help for clinicians as well as for patients, ultimately facilitating treatment and resulting in a shorter time to pregnancy and live birth.

## Author contributions

SE designed the manuscript. All authors contributed to drafting and critical discussions. GB and MR scrutinized the literature and developed the Tables. All authors contributed to revised and accepted the final manuscript.

### Conflict of interest statement

SE received honoraria for lectures from Merck, Besins, and Lilly. MR received honoraria for lectures from Merck. GB and AC have nothing to disclose. PH received unrestricted research grants from MSD, Merck, and Ferring as well as honoraria for lectures from MSD, Merck and IBSA. CA received honoraria for lectures from Merck. The reviewer NP declared a past co-authorship with several of the authors to the handling Editor.
